# BSA-seq Identifies a Major Locus on Chromosome 6 for Root-Knot Nematode (*Meloidogyne graminicola*) Resistance From *Oryza glaberrima*

**DOI:** 10.3389/fgene.2022.871833

**Published:** 2022-06-14

**Authors:** Gurwinder Kaur, Inderjit Singh Yadav, Dharminder Bhatia, Yogesh Vikal, Kumari Neelam, Narpinderjeet Kaur Dhillon, Umesh Preethi Praba, Gurjit Singh Mangat, Kuldeep Singh

**Affiliations:** ^1^ School of Agricultural Biotechnology, Punjab Agricultural University, Ludhiana, India; ^2^ Department of Plant Breeding and Genetics, Punjab Agricultural University, Ludhiana, India; ^3^ Department of Plant Pathology, Punjab Agricultural University, Ludhiana, India; ^4^ International Crops Research Institute for the Semi-Arid Tropics (ICRISAT), Hyderabad, India

**Keywords:** BSA-QTLseq, candidate genes, *Oryza glaberrima*, SNPs, *Meloidogyne graminicola*

## Abstract

Root-knot nematode (*Meloidogyne graminicola*) is one of the emerging threats to rice production worldwide that causes substantial yield reductions. There is a progressive shift of the cropping system from traditional transplanting to direct-seeded water-saving rice production that favored the development of *M. graminicola*. Scouting and deploying new resistance genes is an economical approach to managing the root-knot nematodes. Here, we report that the inheritance of root-knot nematode resistance in *Oryza glaberrima* acc. IRGC102206 is governed by a single dominant gene. Traditional mapping coupled with BSA-seq is used to map nematode resistance gene(s) using the BC_1_F_1_ population derived from a cross of *O. sativa* cv. PR121 (S) and *O. glaberrima* acc. IRGC102206 (R). One major novel genomic region spanning a 3.0-Mb interval on chromosome 6 and two minor QTLs on chromosomes 2 and 4 are the potential genomic regions associated with rice root-knot nematode resistance. Within the QTL regions, 19 putative candidate genes contain 81 non-synonymous variants. The detected major candidate region could be fine mapped to accelerate marker-assisted breeding for root-knot nematode resistance in rice.

## Introduction

Rice (*Oryza sativa* L.) is one of the staple food crops that feed half of the world’s population. Its production has been continuously increasing at a constant pace for the last 10 years. However, to meet the demand of the ever-growing population, there is still a need to increase rice production by 2050 (Ray et al., 2013). Over the changing agro-climatic conditions, various biotic and abiotic stresses have emerged that threaten rice production across the globe. Among biotic stresses, plant-parasitic nematodes pose the foremost warning to rice production worldwide (Mhatre et al., 2017). About 300 nematode species of 35 genera infect rice, and the *Meloidogyne* genus is the first among the top 10 plant-parasitic nematodes (Nicol et al., 2011). Within the *Meloidogyne* genus, root-knot nematode (*M. graminicola*) is the most widespread threat nowadays in almost all rice-growing systems—upland, lowland, deepwater, and irrigated rice—and causes significant yield losses ranging from 20% to 80% (Mantelin et al., 2017; Kumar, 2020).

The second stage juveniles (J2) of root-knot nematode penetrate behind the root cap due to the absence of differentiated endodermis near the root tip (Bridge et al., 2005). After penetration, juveniles migrate intercellularly through the root cortex toward the apical meristem and the cellular differentiation region and establish a permanent feeding site in the vascular tissue to develop giant cells (Williamson, 1998). The metabolically active giant cells serve as a source of nutrients for nematodes to complete their life cycle between 19 and 27 days from juveniles to adults and release eggs around the root surface. These nematodes complete numerous generations in a single rice-growing season to build up a high damaging population in a shorter period (Shrestha et al., 2007). The mechanical disruption caused by the giant cells in metaxylem vessels interferes with the uptake of water and nutrients that strongly impair the root physiology and development (Singh, 2010). The disruption of water translocation and nutrient transport by the root vascular system leads to stunting, chlorosis, and loss of vigor, which eventually result in reduced growth and finally cause significant yield losses of the crop (Mantelin et al., 2017).

Some cultural practices are followed to limit the nematode population below a damaging threshold level. Flooding and crop rotation practices are partially effective and of limited use due to the broad host range of *M. graminicola* and an unacceptable cost of non-hosts, such as mung bean, mustard, and sesame, for small-scale farmers using rice as a staple food. The use of nematicides is uneconomic, unhealthy for the environment, and unsafe for human health. Alternatively, host resistance is effective and economical to manage root-knot nematode population densities below the threshold levels and gains significance in water-saving practices during the shifting of rice cultivation from irrigation to direct-seeded rice. Most Asian genotypes are susceptible to root-knot nematode, with only a few of them being resistant (Dimkpa et al., 2016). Natural resistance to *M. graminicola* has been reported in *O. glaberrima* (African rice) and *O. longistaminata* (Soriano et al., 1999). But due to the presence of sterility genes and the low yield potential of *O. glaberrima,* limited efforts have been made to introgress root-knot nematode resistance from *O. glaberrima* into *O. sativa*. However, fertility can be retained by recurring backcrossing for a few generations, but backcrossing for several generations increases the risk of losing desirable traits. Moreover, interspecific progenies do not show a similar type of resistance as *O. glaberrima* (Plowright et al., 1999). Several studies have reported the quantitative nature of resistance against *M. graminicola* as QTLs for root galling and the number of galls and eggs per root system have been identified using RIL populations (Shrestha et al., 2007; Jena et al., 2013; Galeng-Lawilao et al., 2018). In some crop systems, a single major gene confers resistance to different *Meloidogyne* species; for example, *rkn1* confers resistance to *M. incognita* in cotton (Wang et al., 2006), *Mi* from *Lycopersicon peruvianum* (Roberts, 1995) gives resistance to some of the root-knot nematode species in tomato (Abad et al., 2003), and *Hsa-1Og* provides resistance against cyst nematode (*Heterodera sacchari*) in rice (Lorieux et al., 2003). Recently, Mhatre et al. (2017) have reported hypersensitive response (HR) in rice cultivar Zhonghua11 (Asian rice) against *M. graminicola* and suggested that resistance is due to major genes rather than quantitative resistance. There is still a continuous need to explore and exploit related species of rice for resistance to *M. graminicola*.

Identification and characterization of genes/QTLs responsible for root-knot nematode resistance are important not only to unveil the molecular mechanisms of resistance but also to deploy the resistance genes for the development of nematode-resilient rice cultivars. Different molecular mapping strategies have been used to map genes/QTLs for several traits in rice. Bulked segregant analysis (BSA) is one of the effective methods to map genes or QTLs from a population having two extreme phenotypic traits (Michelmore et al., 1991; Venuprasad et al., 2009). Recent development in next-generation sequencing technologies has provided effective tools for genome-wide identification of SNPs and other structural variants, and also genotyping (Huang et al., 2009) has accelerated genetic mapping studies and marker development. A “BSA-seq” approach that couples whole-genome re-sequencing and BSA of extreme phenotypes is cost-effective and rapidly identifies genomic regions associated with a trait of interest (Takagi et al., 2013; Deokar et al., 2019). This approach has been used successfully in different crops such as rice, tomato, chickpea, and brassica to map QTLs of different genetic complexities from single genes to major QTLs from last few years (Takagi et al., 2013; Illa-Berenguer et al., 2015; Deokar et al., 2019; Zhang et al., 2020). There is only one report of QTL identification for rice root-knot nematode resistance through BSA-seq analysis using mapping population derived from indica and aus cultivar (Lahari et al., 2019). The present study aims to 1) determine the genetics of *M. graminicola* resistance in *O. glaberrima*, 2) identify major locus associated with root-knot nematode resistance through BSA-seq, and 3) explore candidate genes conferring *M. graminicola* resistance and identification of SNPs within the candidate genes.

## Materials and Methods

### Plant Materials

The plant material consisted of *O. glaberrima* acc. IRGC102206, PR121, BC_1_F_1_, BC_2_F_1_, and BC_2_F_2_ populations derived from the cross of PR121 and *O. glaberrima* acc. IRGC102206 *O. glaberrima* acc. IRGC102206 were identified as highly resistant and susceptible to *M. graminicola*, respectively, in a previous study (Kaur, 2020). The resistance was confirmed over 2 years of screening both in the nematode-infested pots and sick plot under field conditions, as the number of galls per plant was significantly lower as compared to susceptible checks. *O. glaberrima* has characteristics of early maturity, moderate to tall height, seed shattering, lower yield, and more resistance to various diseases and pests. However, PR121 has short stature, bacterial blight resistance, and better lodging tolerance.

### Phenotypic Evaluation of Backcross Generations for Nematode Infestation

The BC_1_F_1_, BC_2_F_1_, and BC_2_F_2_ populations were grown in the nursery during cropping seasons 2018–2020. The 25-day-old seedlings were transplanted in irrigated field conditions with plant-to-plant and row-to-row distances of 20 and 30 cm, respectively. Each plant of BC_1_F_1_, BC_2_F_1_, and BC_2_F_2_ after 15 days of transplanting was split into four plantlets, and three replicas of each BC_1_F_1_, BC_2_F_1_, and BC_2_F_2_ plant were transferred to a nematode-infested sick plot with an initial population density of 1 J2/g of soil while one replica was raised under non-infested conditions. Standard agronomic practices were implemented during the raising of the crop, except that the soil was not flooded during screening in the nematode-infested sick plot. Each plant of all populations was uprooted from the nematode-infested sick plot after 60 days of transplanting. The roots were washed immediately under running tap water to count the galls per root system. Root gall index was calculated on a scale of 1–5 as given by Gaur et al. (2001). Rating was done as follows: 1 for 0–1 gall (highly resistant), 2 for 1–10 galls (resistant), 3 for 11–30 galls (moderately resistant), 4 for 30–100 galls (susceptible), and 5 for >100 galls (highly susceptible). The segregation pattern for nematode resistance was checked in each generation using standard chi-square analysis for the goodness of fit. Data for each BC_1_F_1_ plant on different morphological characters like plant height (cm), root length (cm), fresh shoot weight (g), and fresh root weight (g) were measured immediately after the uprooting of the plants. The shoots and roots of each plant were packed separately in brown paper bags for drying, to achieve constant weight for measuring dry shoot and root weight (g). Data were analyzed using a generalized linear model (GLM) of SAS software version 9.4 (SAS Institute, Cary, NC, United States).

### Traditional Rough Mapping

Genomic DNA was isolated from the young leaves of parents and each BC_1_F_1_ plant using the CTAB method (Doyle and Doyle, 1987). The degradation and contamination of DNA were checked on 0.8% agarose gel while DNA was quantified using Thermo scientific NanoDropTM 1000 spectrophotometer. A total of 512 simple sequence repeat (SSR) markers from the universal core genetic map (Orjuela et al., 2010) spanning all 12 rice chromosomes were used for the parental polymorphic survey on PR121 and *O. glaberrima* acc. IRGC102206. Primer sequences were retrieved from the Gramene database (http://www.gramene.org/; IRGSP, 2005). *In vitro* amplification using polymerase chain reaction (PCR) was performed in a 96-well PCR plate in Eppendorf and Applied Biosystems master cyclers. The total PCR reaction of 20 μl was prepared using the following components: 100 ng template DNA, 0.50 μM each of forward and reverse primers and 2× Emerald Amp^®^ GT PCR Master Mix containing an optimized buffer, PCR enzyme, dNTP mixture, gel-loading dye (green), and a density reagent. A negative control (without template DNA) was included in each amplification reaction. PCR profile of 95°C for 5 min, followed by 30 cycles of 1 min at 94°C, 1 min at 55–60°C, and 1 min at 72°C with a final extension of 10 min at 72°C was used for amplification. The amplified products were resolved in 3.0% agarose gel, and amplicons were scored by comparing them to parental alleles. The linkage map was constructed using the Kosambi mapping function of QTL IciMapping version 4.1 (Meng et al., 2015) through MAP functionality in a graphic form representing the position of markers within linkage groups by using a threshold LOD score of 3.0. Composite interval mapping (CIM) at a 95% threshold level was used for the identification of QTLs based on 1,000 permutation tests using Windows QTL cartographer version 2.5 (Wang et al., 2012). The position of putative QTLs corresponded to the location (in centiMorgans) of peak LOD scores in the scan of individual chromosomes and was designated according to the chromosome position. The proportion of observed phenotypic variance attributable to the QTL was estimated by the coefficient of determination (*R* ^2^) using the maximum likelihood of CIM.

### Bulked Segregant Analysis Through Whole-Genome Re-Sequencing

**FIGURE 1:**
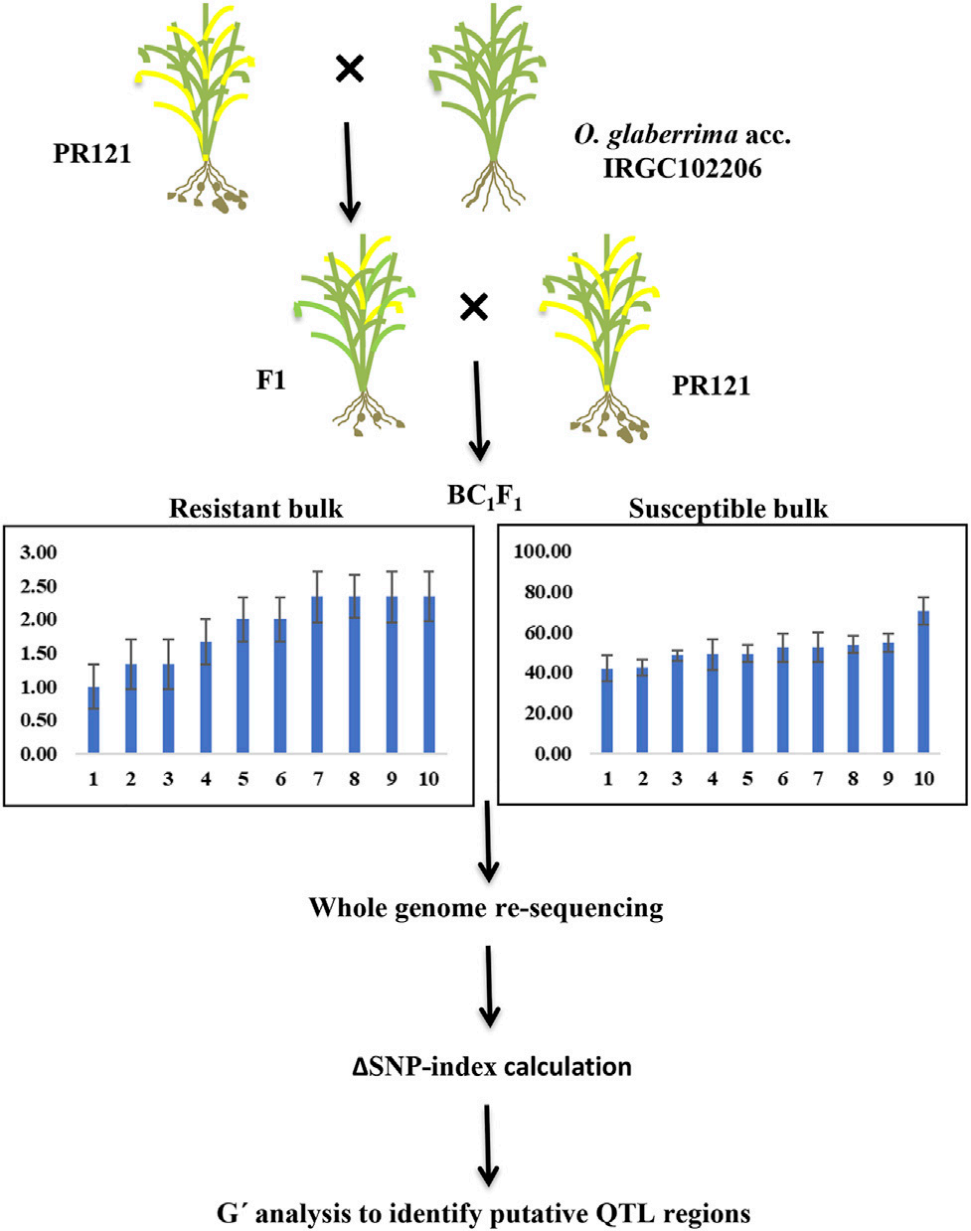
Flow chart for BSA-seq using BC_1_F_1_ individuals derived from the cross of PR121 (S) and *O. glaberrima* acc. IRGC102206 (R).

The DNA concentration of each sample was normalized to 500 ng/μl, and 5 μg of the total DNA of the individual plant was used for making extreme bulks. An equal amount of DNA from 10 individual plants with few galls (1–2.33 galls) was mixed to generate the resistant DNA bulk. Similarly, an equal amount of DNA from 10 individual plants with higher gall numbers per plant (40–75 galls) was mixed to generate the susceptible DNA bulk (Figure 1). Paired-end sequencing libraries from the two extreme bulk and parents using 2 μg of DNA were prepared according to the Illumina manufacturer’s instructions. The sequencing of libraries using the Illumina HiSeqTM 2500 platform was outsourced from NGB Diagnostics Pvt Ltd., India. Illumina sequencing of genomic libraries for each of the parents and the two bulks (2 × 150 bp) produced 33–35 million reads per sample for a total of 136 million raw reads. The quality of raw reads was assessed using FASTQC (version 0.11.8; Andrews, 2010) with default parameters. Poor-quality sequences were filtered and removed while contaminated adapter sequences and any unwanted bias from their ends were trimmed using Trimmomatic (version 0.39; Bolger et al., 2014). A Phred score of 30 was kept as the overall quality threshold for raw reads. The filtered reads were further re-checked for quality using FASTQC.

### BSA-seq Analysis

High-quality sequences were aligned and mapped to the *Oryza sativa* Indica Group ASM465v1 reference sequence of cultivar 93-11, available at Ensembl plants (https://plants.ensembl.org/Oryza_indica), using Bowtie 2 algorithm with default parameters (version v2.0.0; Langmead and Salzberg, 2012). The Bowtie 2 default mode is faster than all Burrows-Wheeler Aligner (BWA) modes and more than 2.5 times faster than the BWA default mode. All Bowtie 2 modes aligned a greater number of reads than either BWA or short oligonucleotide alignment program 2 (SOAP2). To keep only uniquely mapping reads, the output SAM files were converted into BAM files, then read groups were added, sorted, and indexed using SAMtools (version 0.1.19; Li et al., 2009). The output BAM files containing uniquely mapped reads were used for SNP Calling through the GATK (Genome Analysis Toolkit) pipeline. Subsequently, GATK’s HaplotypCaller component (version 4.0; Mckenna et al., 2010) was used to perform a joint variant calling of all samples. The indels and missing data were filtered out using the variant filtration parameter of vcftools (https://github.com/vcftools/vcftools). The filtered VCF file in table format was used as an input file for QTLseqr (https://github.com/bmansfeld/QTLseqr) package developed by Mansfeld and Grumet (2018). SNPs with a reference allele frequency of 0.2 from both the bulks were filtered out as these might be due to sequencing or alignment error. In G′ approach, run GprimeAnalysis first counted the number of SNPs within the sliding window, and then a tricube-smoothed ∆SNP index was calculated within a window size of 1.0-Mb genomic region. The ∆SNP index (>0.1) was used to calculate *p*-values (<0.05) and an FDR(*q*) of 0.01 to identify potential QTLs associated with root-knot nematode resistance. The G′ determines the statistical significance of QTLs as background noise is less and also addresses the linkage disequilibrium (LD) between SNPs. One important advantage of this method is that *p*-values can be estimated for each SNP using non-parametric estimation of the null distribution of G′ (Magwene et al., 2011). Comparison of the QTL-seq method (Delta-SNP index) and G′ method by Mansfeld and Grumet (2018) showed that a confidence interval of 99% with the QTL-seq method was not as stringent as using an FDR of 0.01 in the G′ method. All commands and codes for BSA-seq analysis are available at https://github.com/bmansfeld/QTLseqr.

### Identification of Candidate Genes

The QTL regions harboring the candidate genes based on the annotation of *Oryza sativa* indica Group ASM465v1 (https://plants.ensembl.org/Oryza_indica) were identified. To identify non-synonymous SNPs among the candidate genes of the two parents (PR121 and *O. glaberrima*), nucleotide changes were investigated using ExPASy translate tool (http://web.expasy.org/translate/). Potential candidate genes and their corresponding Ensembl IDs were further subjected to the ShinyGO v0.74 database (Ge et al., 2019) to obtain gene ontology (GO) annotation against *O. sativa* subsp. indica. GO enrichment was calculated by a *p*-value cut-off (FDR) at 0.05 for the genes.

## Results

### Inheritance of Root-Knot Nematode Resistance

**FIGURE 2:**
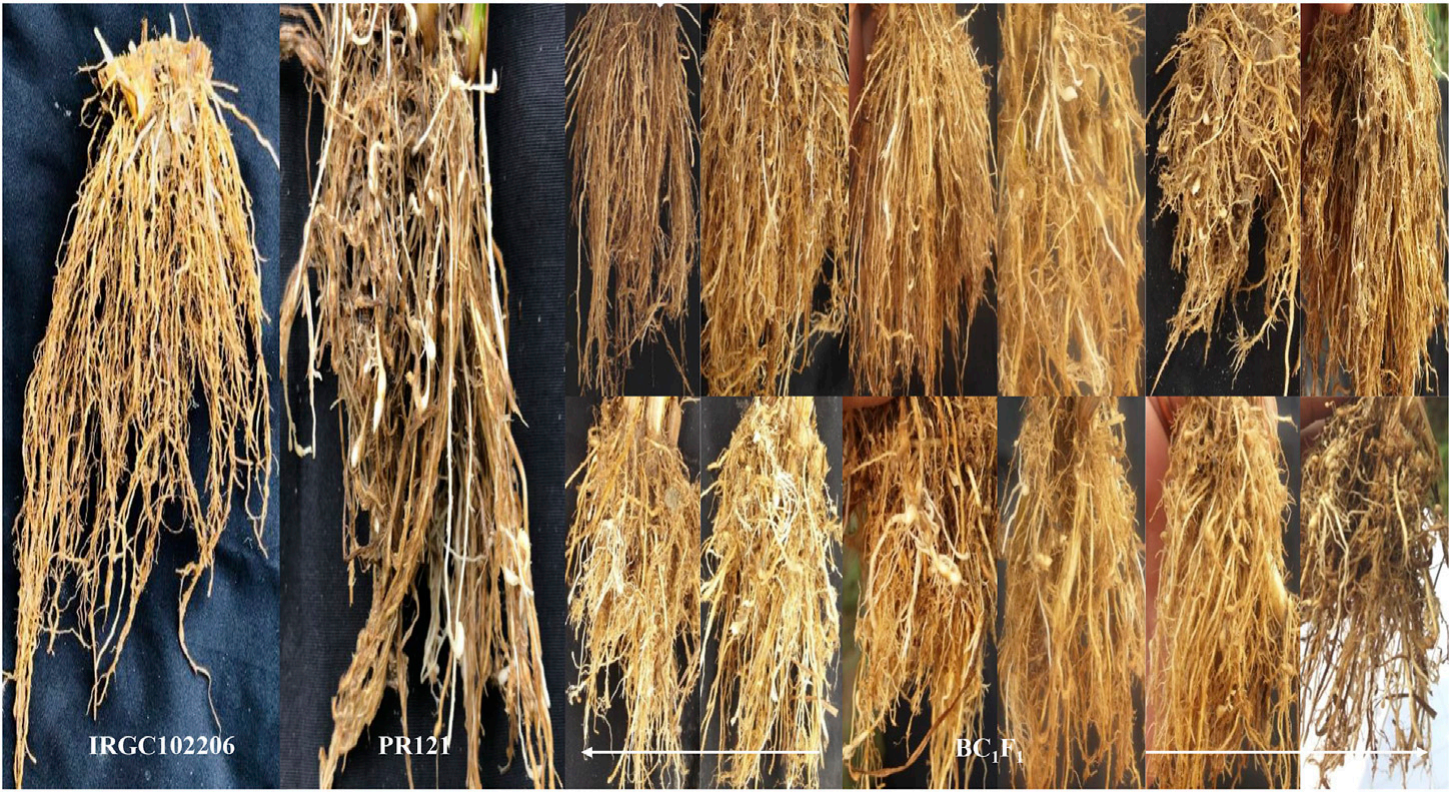
Response of *O. glaberrima* acc. IRGC102206 (R), PR121 (S), and their derived BC_1_F_1_ progenies upon *M. graminicola* infestation.

The F_1_s (with medium-sized ligule) generated from the cross of PR121 (S) and *O. glaberrima* acc. IRGC102206 (R) were partial to completely sterile; therefore these F_1_s were backcrossed with PR121 to develop the BC_1_F_1_ population. A total of 10,800 spikelets of F_1_ plants were cross-pollinated and a 0.95% seed setting of BC_1_F_1_ (103 seeds) was obtained. Out of the 103 seeds, 69 seeds (67%) were germinated in the nursery and transplanted in controlled (normal) conditions. After 25 days of transplanting, three replicas of each BC_1_F_1_ plant along with their parental genotypes were screened in the nematode-infested sick plot. Based on gall number, 39 and 30 BC_1_F_1_ plants were categorized as resistant and susceptible, respectively, for root-knot nematode resistance that corresponded to a single locus segregation ratio statistically (Table 1). The resistant parent IRGC102206 showed gall number from 0 to 1 while the susceptible parent PR121 exhibited gall number in the range of 64–82 (Figure 2). The average gall number and gall index among BC_1_F_1_ individuals ranged from 1.00 to 70.33 and 1.33 to 4.00, respectively (Table 2). The resistant parent *O. glaberrima* accession IRGC102206 had an overall mean gall index of 1.0 while the susceptible parent PR121 had a mean gall index of 4.0. The segregation pattern in successive generations, that is, BC_2_F_1_ and BC_2_F_2_, was authenticated, thereby confirming that nematode resistance is governed by a single dominant gene (Table 1). Overall, BC_1_F_1_ plants showed stunted growth under nematode infestation. Plant height and root length among BC_1_F_1_ plants ranged from 61 to 118 cm and 7.57 to 21.03 cm, respectively. Significant differences were observed among BC_1_F_1_ plants for all the traits (Table 2). There was a nominal decrease in growth parameters in the resistant individuals compared to the susceptible plants indicating that the plant growth parameters were affected by nematode infestation.

**TABLE 1:** Genetic analysis of root-knot nematode resistance in different generations derived from the cross of PR121 (S) and *O. glaberrima* acc. IRGC102206 (R).

Generation	Total no. of plants analyzed	No. of resistant plants	No. of susceptible plants	Expected segregation ratio	χ^2^	*p*-value
BC_1_F_1_	69	39	30	1:1	1.16	0.28^ns^
BC_2_F_1_	276	154	122	1:1	3.7	0.05^ns^
BC_2_F_2_	231	175	56	3:1	0.07	0.79^ns^

**TABLE 2:** Means of growth parameters, gall number, and gall index of parents and BC_1_F_1_ population derived from the cross of PR121 (S) and *O. glaberrima* acc. IRGC102206 (R) in nematode-infested conditions.

Parents/population	Plant height (cm)	Root length (cm)	Fresh shoot weight (g)	Fresh root weight (g)	Dry shoot weight (g)	Dry root weight (g)	Gall number	Gall index
PR121	57.83 ± 0.1	11.67 ± 1.4	58.36 ± 1.9	30.95 ± 0.4	8.09 ± 1.5	4.70 ± 0.6	73.66 ± 5.23	4.00 ± 0.0
IRGC102206	101.16 ± 3.1	28.67 ± 0.5	112.93 ± 2.3	45.03 ± 0.4	20.73 ± 0.3	11.81 ± 0.3	0.67 ± 0.3	1.00 ± 0.0
BC_1_F_1_	87.00 ± 9.2 (61.0–118.0)^b^	14.30 ± 1.7 (7.57–21.03)	110.60 ± 23.1 (14.89–188.48)	41.50 ± 12.9 (6.01–84.42)	18.60 ± 5.8 (3.99–38.28)	8.40 ± 2.5 (2.37–17.81)	23.20 ± 10.8 (1.00–70.33)	3.00 ± 0.5 (1.33–4.00)
CV^a^	7.54	15.26	11.39	16.63	21.68	31.59	29.49	14.23
*F* value	15.99^c^	3.85^c^	28.65^c^	30.16^c^	16.81^c^	6.64^c^	20.86^c^	13.67^c^

a CV, coefficient of variance.

b Value in parentheses indicates the range of a trait in BC_1_F_1_ population.

c Indicates significant level at *p* < 0.01.

### Traditional Mapping

We also carried out rough QTL mapping using 69 individuals of the BC_1_F_1_ population. A total of 512 microsatellite markers spanning 12 chromosomes of rice were used for the parental polymorphic survey and the markers per chromosome varied from 53 (chromosomes 1 and 2) to 31 (chromosome 9). Parental polymorphism among all chromosomes ranged from 7.6% to 50.9% with an average of 31.25%. A comprehensive list of polymorphic SSR markers is given in Supplementary Table S1. A total of 100 polymorphic SSR markers were genotyped and a genetic linkage map was generated with a total map length of 1,901.21 cM, with an average distance of 22.63 cM. One putative QTL associated with gall numbers was detected on chromosome 6 designated as *qGN6.1*, between the marker interval of RM3183 and RM27001 explaining the phenotypic variance of 41% at an LOD score of 3.95 (Table 3). Two QTLs for dry root weight and dry shoot weight (*qDRW3.1* and *qDRW3.2*) were co-localized on chromosome 3 whereas QTL for fresh root weight, *qFRW8.1*, was located on chromosome 8 with 17% of total phenotypic variance (Table 3).

**TABLE 3:** Chromosomal locations and parameters associated with the quantitative trait loci (QTL) for resistance to rice root-knot nematode in BC_1_F_1_ population derived from the cross of PR121 (S) and *O. glaberrima* acc. IRGC102206 (R).

Trait	QTLs^a^	Flanking markers	Physical position (Mb)	LOD score^b^	PVE^c^ (%)	AE^d^
Gall number	*qGN6.1*	RM3183-RM20071	12.29–16.36	3.95	41.90	−23.62
Fresh root weight	*qFRW8.1*	RM23174-RM210	21.07–22.46	3.4	17.43	35.70
Dry root weight	*qDRW3.1*	RM3204-RM15281	14.82–18.48	3.1	22.67	−5.60
	*qDRW3.2*	RM5626-RM168	24.67–27.89	4.8	25.40	6.29
Dry shoot weight	*qDSW3.1*	RM3204-RM15281	14.82–18.48	2.6	18.71	−11.46
	*qDSW3.2*	RM5626-RM168	24.67–27.89	4.1	21.72	11.98

a Putative QTLs are designated by the corresponding chromosome in which they are found. The method described by McCouch et al. (1997) was followed for QTL nomenclature.

b The maximum LOD score associated with each QTL.

c *R* ^2^ estimates the proportion of phenotypic variance (%) explained by the detected QTL.

d The additive genetic effect of the putative QTL. A negative number indicates that the alleles for resistance are derived from the male donor parent (*O. glaberrima* acc IRGC102206) and a positive number means that the alleles are contributed by the female parent (PR121).

### Whole-Genome Re-Sequencing of Bulked Segregant Analysis Pools

Based on the gall number of BC_1_F_1_ population, 10 plants from each of the extreme values of frequency distribution were selected and pooled as resistant bulk (RB) and susceptible bulk (SB), respectively. The whole-genome re-sequencing data from PR121, IRGC102206, resistant, and susceptible bulks were aligned with the *O. sativa* cultivar 93-11 reference genome using Bowtie 2 algorithm with default parameters. A total of 35.0, 33.6, 34.3, and 34.0 million paired-end reads were generated from IRGC102206, PR121, RB, and SB, respectively (Table 4), and 32–34 million reads of paired-end sequences were retained which were equivalent to 12.8× to 14.9× coverage of the rice genome indicating the high quality of the sequencing data (Supplementary Figure S1). Approximately, 31.23 (96.11%), 29.78 (87.66%), 31.07 (93.33%), and 31.05 (93.86%) million read pairs of PR121, IRGC102206, RB, and SB were uniquely mapped to the reference genome, respectively (Table 4). The GC content of raw reads ranged from 42% to 43% for all the samples.

**TABLE 4:** Statistical summary of BSA-seq data of parental lines, resistant bulk, and susceptible bulk.

Parameters	PR121	*O. glaberrima* acc. IRGC102206	Resistant bulk	Susceptible bulk
Total sequenced reads	33,626,673	35,036,905	34,300,698	34,021,906
High-quality reads (Q30)	32,496,792 (96.63%)	33,982,343 (96.99%)	33,298,078 (97.07%)	33,082,740 (97.23%)
Low-quality reads	1,129,881 (3.37%)	1,054,562 (3.01%)	1,002,620 (2.93%)	939,166 (2.77%)
Uniquely mapped reads	31,233,034	29,789,156	31,077,973	31,052,534
Alignment rate (%)	96.12	87.71	93.35	93.89
Average depth	14.9	12.8	12.5	12.5

A total of 3,692,066 variants (SNPs/indels) were identified by a joint variant calling with the reference genome through the GATK pipeline. RB had the highest variant (1,641,906) followed by IRGC102206 (1,049,225), PR121 (695,856), and SB (305,079). Maximum SNPs (284,012) were specific to chromosome 1 while chromosome 11 had the least SNPs (123,492). Likewise, the variant rate (SNPs/Mb) also varied among chromosomes, and chromosome 6 possessed the highest variant rate (1 SNP/195 bases) while the lowest variant rate (1 SNP/136 bases) was detected for chromosome 7 (Supplementary Table S2). A total of 1,416,115 SNPs were detected between RB and SB. Based on reference allele frequency (0.20) and maximum total depth (300), 174,651 SNPs were used for QTL identification through BSA-seq analysis.

### BSA-seq Identifies Root-Knot Nematode Resistance Major Locus on Chromosome 6

**FIGURE 3:**
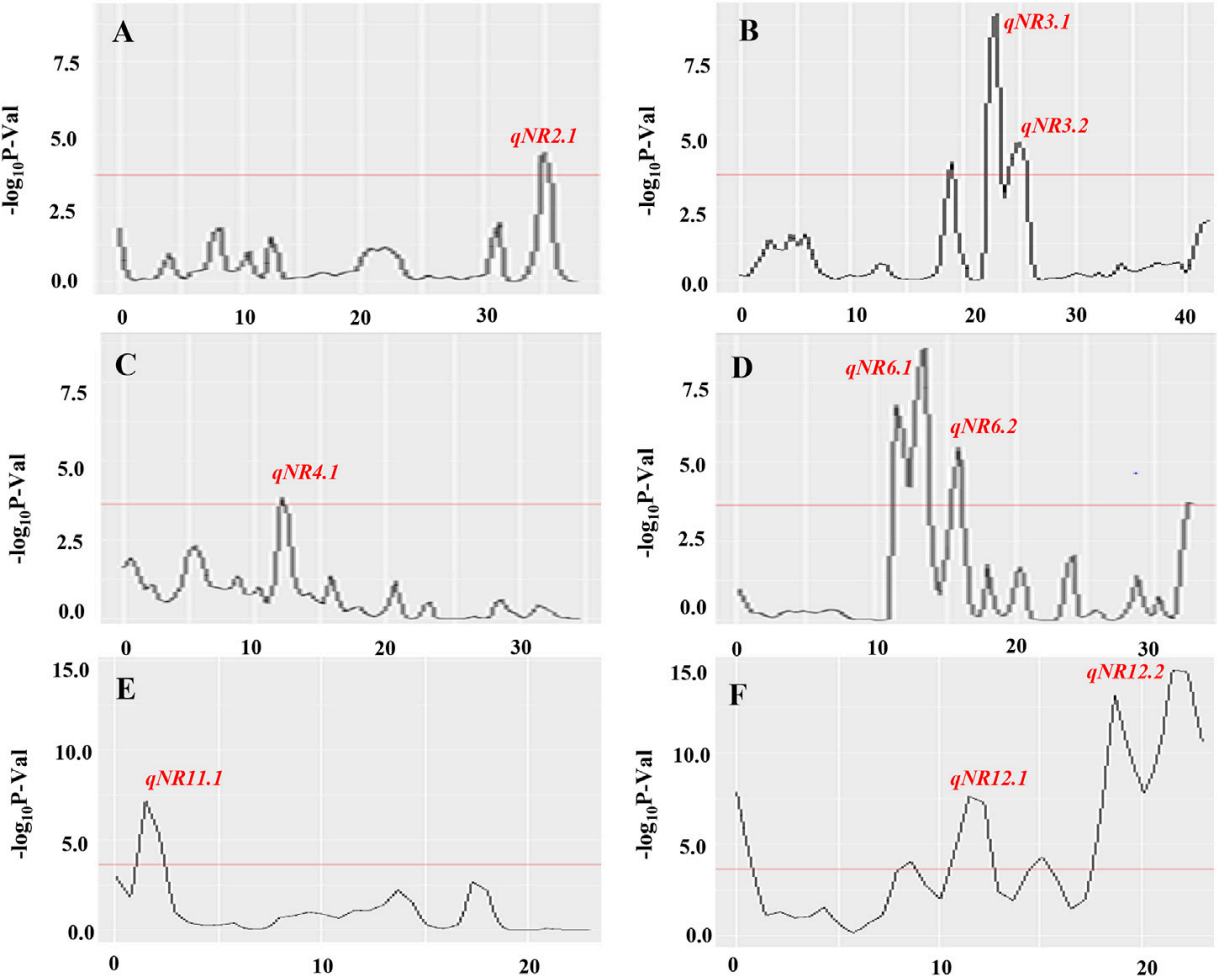
Quantitative trait loci for root-knot nematode resistance identified on chromosomes 2 **(A)**, 3 **(B)**, 4 **(C)**, 6 **(D)**, 11 **(E)**, and 12 **(F)** using BSA-seq analysis. Distribution of –log10 *p*-value was calculated within a 1.0-Mb sliding window using tricube-smoothed kernel. The *Y*-axis represents – log_10_ *p*-values and the *X*-axis represents the position of chromosomes in Mb. The red line represents the significance threshold for FDR = 0.01, and the genomic region where –log10 *p*-value crosses the threshold was considered as significant QTL. Out of 12 chromosomes, significant QTLs identified on six chromosomes are shown.

The BSA-seq analysis detected nine putative QTL regions on chromosomes 2, 3, 4, 6, 11, and 12, based on the calculation of Gʹ values of SNP within a window size of 1.0-Mb genomic region across the entire length of chromosomes. QTLs on chromosomes 3, 6, and 12 with large G′ peaks above FDR(*q*) of 0.01 were considered as major QTL regions responsible for root-knot nematode resistance in rice. Despite the major G′ peaks, five minor G′ peaks at the margin of significance have been identified on chromosomes 2, 3 (left to *qNR*3.1), 4, and 12 (on both sides of *qNR12.1*). Two adjacent QTLs on chromosome 3, namely, *qNR3.1* and *qNR3.2*, had peaked in genomic intervals of 22.00–23.3 Mb and 24.0–25.6 Mb, respectively (Figure 3B). Similarly, QTLs on chromosome 12, designated as *qNR12.1* and *qNR12.2*, were located at 5 Mb distance between intervals of 10.5–12.7 Mb and 17.5–22.9 Mb, respectively. *qNR12.2* was present at the proximal region on the long arm of chromosome 12 displaying two adjacent component peaks (Figure 3F). The region covered by significant QTLs varied from 0.2 Mb (*qNR4.1*) to 5.4 Mb (*qNR2.2*). The direction of the ΔSNP index value and the allele frequency difference (AFD) indicated that *qNR2.1*, *qNR4.1*, *qNR6.1*, and *qNR6.2* originated from the donor parent IRGC102206 (Table 5). However, QTLs on chromosomes 3, 11, and 12 originated from a susceptible parent, PR121. The number of SNPs present in *qNR3.1, qNR3.2, qNR11.1*, *qNR12.1*, and *qNR12.2* region was 192, 330, 328, 691, and 322, respectively.

**TABLE 5:** List of QTLs identified through BSA-seq for rice root-knot nematode (*M. graminicola*) resistance.

Chromosome	QTL^a^	Start (Mb)	End (Mb)	Interval (Mb)	*p*-value	AFD^b^
2	*qNR2.1*	34.82	35.52	0.70	9.89E-05	0.29
3	*qNR3.1*	22.04	23.37	1.33	1.22E-05	−0.37
3	*qNR3.2*	24.05	25.61	1.56	6.11E-05	−0.29
4	*qNR4.1*	11.90	12.14	0.24	0.008847	0.33
6	*qNR6.1*	11.28	13.68	2.40	7.60E-06	0.18
6	*qNR6.2*	15.48	16.09	0.61	2.61E-05	0.74
11	*qNR11.1*	1.01	2.38	1.36	5.33E-06	−0.52
12	*qNR12.1*	10.52	12.73	2.20	1.92E-05	−0.20
12	*qNR12.2*	17.55	22.99	5.43	5.80E-06	−0.69

a Putative QTLs are designated by the corresponding chromosome in which they are found. The method described by McCouch et al. (1997) was followed for QTL nomenclature.

b Allele frequency difference.

The genomic interval between *qNR6.1 and qNR6.2* was 2.4 Mb (11.2–13.6 Mb) and 0.6 Mb (15.4–16.0 Mb), respectively. The main Gʹ peak exhibited a subpeak in the QTL, *qNR6.1*, region whereas *qNR6.2* exhibited a clear sharp peak region and fall near the centromere region (ranging approximately 17.00–22.59 Mb) of the short arm of chromosome 6. These two QTLs must be in continuity suggesting nematode resistance locus spanned within 3.0-Mb regions indicating a significant association for nematode resistance and hence considered as the major locus. The peaks of *qNR2.*1 and *qNR4.1* were at the margin of significance, that is, *qNR2.1* peak slightly exceeding and *qNR4.1* nearly reaching the threshold; and, therefore, referred to as minor QTLs. The number of SNPs present in *qNR2.1, qNR4.1, qNR6.1*, and *qNR6.2* regions was 345, 103, 264, and 27, respectively. Among all QTLs, *qNR6.1* and *qNR12.2* showed the highest Gʹ peaks that greatly exceeded the threshold level indicating the candidate regions for root-knot nematode resistance and susceptibility, respectively. Also, the genomic interval for root-knot nematode resistance on chromosome 6 overlapped with the identified gall number QTL (*qGN6.1*) through SSR markers. Therefore, *qNR6.1* and *qNR*6.2 were selected as promising QTL regions originating from the donor parent for the identification of candidate genes and their sequence variations allied with root-knot nematode resistance.

### Candidate Genes Present Within the Identified QTLs

**FIGURE 4:**
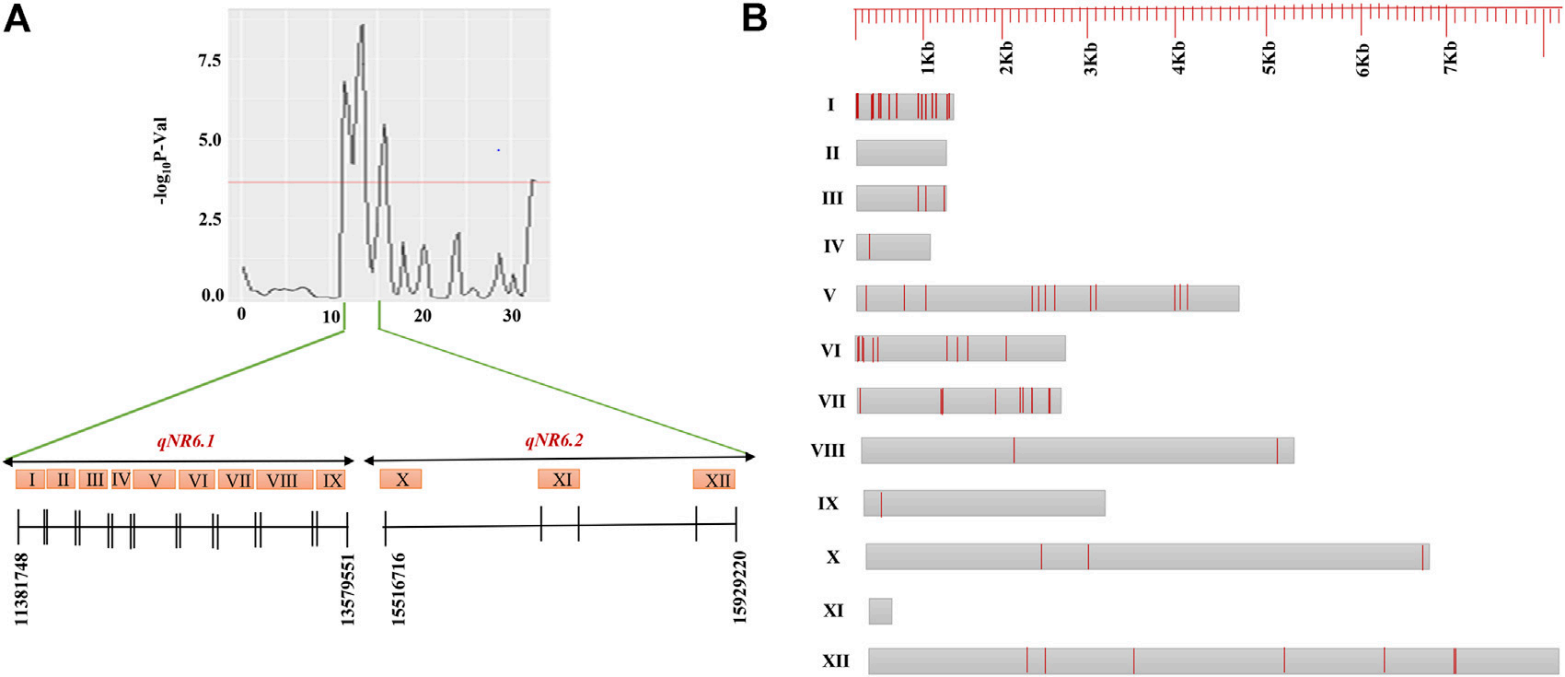
Candidate genes identified in QTLs, *qNR6.1* (I–IX) and *qNR6.2* (X–XII) **(A)**, and intronic and missense variants identified along the total length of genes **(B)**. Gray boxes represent the candidate genes and red lines represent the number of intronic and missense variants in genes.

**FIGURE 5:**
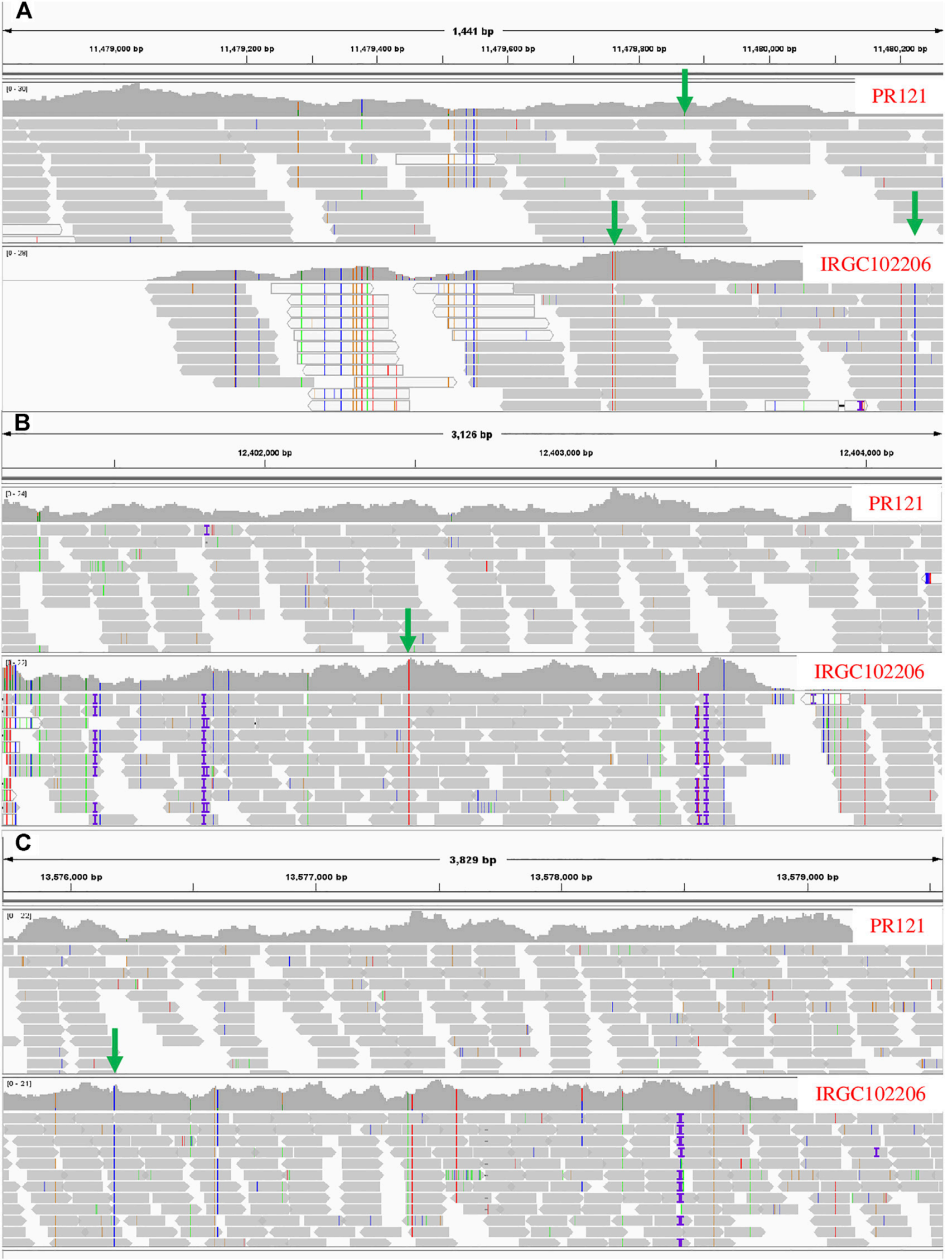
Integrative Genome Viewer showing glycosyltransferase (BGIOSGA022727) **(A)**, peroxidase (BGIOSGA022766) **(B)**, and thioredoxin reductase (BGIOSGA021328) **(C)** candidate genes in the susceptible parent (PR121) and the resistant parent (IRGC102206). The non-synonymous SNPs are highlighted with green arrows.

The candidate genes within the QTL regions were identified based on BGI annotation. The genomic interval of candidate genes and their annotation have been summarized in Supplementary Table S3. The total number of candidate genes annotated in QTLs, *qNR3.1*, *qNR3.2*, *qNR11.1*, *qNR12.1,* and *qNR12.2*, was 7, 10, 16, 10, and 66, respectively. A total of nine annotated genes were identified within a 2.4-Mb interval of *qNR6.1* and three genes within 0.6-Mb interval of *qNR6.2*. The less number of annotated genes within the QTL (*qNR6.1* and *qNR6.2*) interval is because of low recombination rate in the centromere region. In the *qNR2.1* region, seven genes were annotated whereas none of the candidate genes was annotated in the 240-Kb region of *qNR*4.1. We emphasized on those QTLs which originated from the donor parent to identify non-synonymous SNPs in the candidate genes. Among the nine annotated genes of *qNR6.1*, eight genes contained a total of 55 non-synonymous SNPs. In the candidate region of *qNR6.2*, a total of 10 non-synonymous SNPs were detected (Figure 4). The candidate gene encoding glycosyltransferase (BGIOSGA022720) had a maximum number of non-synonymous substitutions by SNPs (15), while peroxidase (BGIOSGA022766) and thioredoxin reductase (BGIOSGA021328) had the least number of non-synonymous SNPs (1). The examination of sequence reads of peroxidase and thioredoxin reductase on Integrative Genome Viewer (IGV) showed that non-synonymous SNPs were specific to the resistant parent; however, the susceptible parent also shared identical alleles to the reference genome (Figure 5). The majority of SNPs have been identified in the coding regions of candidate genes; however, mitogen-activated protein kinase (BGIOSGA022897), fructose-6-P-1-phosphotransferase subunit alpha (BGIOSGA022810), peroxidase (BGIOSGA022766), and thioredoxin reductase (BGIOSGA021328) had intronic SNPs (Table 6). Comparison of amino acid sequences of glycosyltransferase (BGIOSGA021421) and auxin-responsive protein (BGIOSGA022887) revealed that none of the SNPs caused non-synonymous substitutions.

**TABLE 6:** Identification of non-synonymous SNPs in candidate genes present in putative genomic regions associated with rice root-knot nematode resistance.

QTL	Gene	SNP position (bp)	Reference allele	Alternate allele	Variant types	RB bulk variant rate	SB bulk variant rate
*qNR2.1*	BGIOSGA005507	34853274	G	C	Intronic	0.11	0.13
	BGIOSGA009139	35056763	G	A	Intronic	0.25	0.00
		35057030	T	C	Intronic	0.13	0.07
		35058165	C	A	Intronic	0.25	0.33
		35058435	T	C	Intronic	0.40	0.00
		35058636	T	C	Missense	0.25	0.25
		35058713	A	T	Intronic	0.42	0.41
		35058871	A	T	Intronic	0.30	0.07
	BGIOSGA005479	35345082	C	T	Missense	0.55	0.09
		35345096	G	T	Missense	0.47	0.10
		35345396	G	A	Intronic	0.47	0.40
		35345462	T	C	Missense	0.16	0.36
		35345484	C	G	Missense	0.27	0.47
		35346247	C	G	Missense	0.14	0.27
		35346258	T	G	Missense	0.30	0.65
		35346834	C	G	Missense	0.30	0.08
*qNR6.1*	BGIOSGA022720	11381794	C	G	Missense	1.00	0.87
		11381802	T	C	Missense	1.00	0.87
		11382081	T	C	Missense	0.37	0.80
		11382117	G	T	Missense	0.81	0.84
		11382178	A	G	Missense	0.75	0.86
		11382182	C	G	Missense	0.75	0.83
		11382328	C	T	Missense	0.41	1.00
		11382408	A	G	Missense	0.66	0.87
		11382674	A	G	Missense	0.50	1.00
		11382732	G	A	Missense	0.60	1.00
		11382765	C	A	Missense	0.18	0.00
		11382976	T	C	Missense	0.77	1.00
		11382982	T	C	Missense	0.77	1.00
		11383183	T	A	Missense	0.00	0.40
		11383201	T	C	Missense	0.88	0.66
	BGIOSGA022727	11479760	G	T	Missense	0.06	0.21
		11479871	G	A	Missense	0.75	0.80
		11480223	G	A	Missense	0.00	0.15
	BGIOSGA022766	12402483	G	T	Intronic	0.00	0.11
	BGIOSGA022770	12472994	A	C	Missense	0.20	0.37
		12473491	T	C	Missense	0.00	0.33
		12473749	C	T	Intronic	0.50	0.28
		12475176	C	G	Intronic	0.30	0.45
		12475239	G	T	Intronic	0.47	0.44
		12475312	G	A	Intronic	0.57	0.30
		12475415	G	A	Missense	0.52	0.70
		12475767	C	T	Missense	0.20	0.25
		12475832	G	C	Intronic	0.18	0.00
		12476695	C	T	Missense	0.33	0.50
		12476833	A	G	Missense	0.20	0.40
		12476962	A	G	Missense	0.15	0.20
	BGIOSGA022773	12525394	G	A	Missense	0.15	0.20
		12525408	A	G	Missense	0.20	0.23
		12525480	G	T	Missense	0.00	0.09
		12525513	C	A	Missense	0.10	0.08
		12525640	A	C	Intronic	0.10	0.11
		12525681	G	A	Intronic	0.00	0.25
		12526681	G	A	Missense	0.12	0.00
		12526824	C	T	Missense	0.20	0.00
		12526962	T	C	Missense	0.14	0.00
		12527407	C	T	Intronic	0.00	0.30
	BGIOSGA022777	12651941	C	T	Missense	0.14	0.00
		12653109	A	G	Intronic	0.00	0.07
		12653113	G	A	Intronic	0.00	0.07
		12653123	G	C	Intronic	0.00	0.07
		12653667	T	G	Intronic	0.12	0.50
		12653967	G	A	Missense	0.00	0.28
		12654024	A	G	Missense	0.12	0.20
		12654207	G	T	Missense	0.11	0.07
		12654222	G	A	Missense	0.14	0.07
		12654401	T	C	Missense	0.75	0.93
		12654402	A	C	Missense	0.16	0.06
	BGIOSGA022810	13507912	C	G	Intronic	0.16	0.00
		13510886	G	A	Intronic	0.16	0.00
	BGIOSGA021328	13576180	A	C	Intronic	0.18	0.00
*qNR6.2*	BGIOSGA021280	15519303	T	A	Intronic	0.16	0.00
		15519833	A	G	Intronic	0.08	0.00
		15523461	T	G	Missense	0.14	0.00
	BGIOSGA022897	15923363	T	A	Intronic	0.12	0.00
		15923557	T	C	Intronic	0.12	0.00
		15924590	A	G	Intronic	0.12	0.00
		15924970	A	C	Intronic	0.11	0.00
		15927100	A	G	Intronic	0.10	0.00
		15928172	G	A	Intronic	0.09	0.00
		15928187	T	G	Intronic	0.18	0.00

**FIGURE 6:**
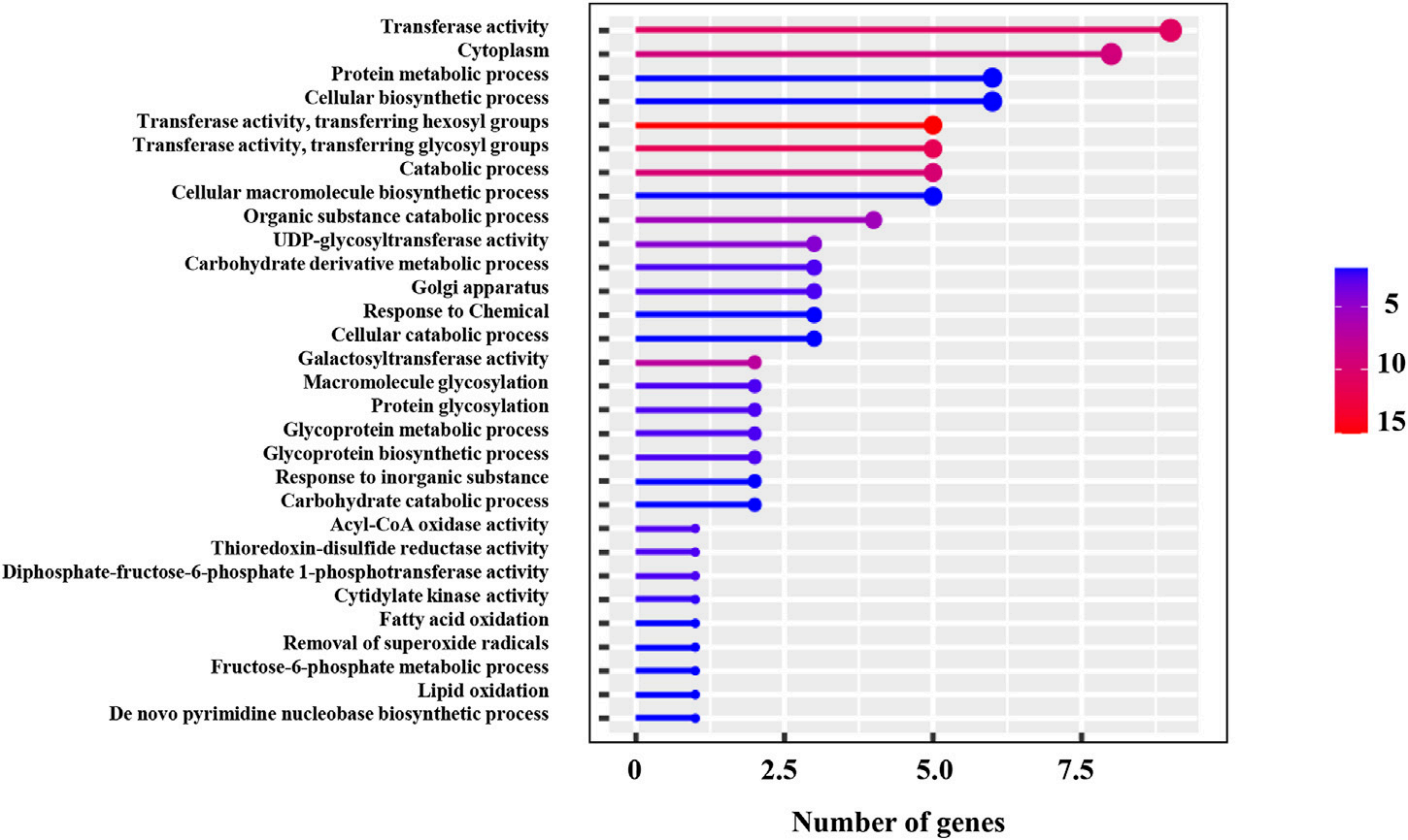
GO annotation of 19 candidate genes identified in *qNR2.1*, *qNR4.1*, *qNR6.1*, and *qNR6.2.*

In the *qNR2.1* region, a total of 85 SNPs were identified within seven candidate genes and only 16 of them had non-synonymous substitution. Among these genes, the miR164 gene (ENSRNA049493468) had no SNP, and none of the SNPs in growth-regulating factor 1 (BGIOSGA005497) and hexosyl transferase (BGIOSGA005471) caused non-synonymous substitutions. Pectin esterase (BGIOSGA005479) genes had 36 synonymous SNPs and 8 non-synonymous SNPs. The maximum non-synonymous SNPs were present in pectinesterase (BGIOSGA005479) followed by a UMP-CMP kinase gene (BGIOSGA009139) among the candidate genes of *qNR2.1* (Table 6). Therefore, candidate genes with non-synonymous SNPs could be the preferred genes for further studies. GO annotation showed the involvement of these genes in various biological processes and molecular functions. The candidate genes were predicted to be involved in different types of biosynthetic, metabolic, and catabolic processes (Figure 6). Transferase activity, protein metabolic process, and catabolic process appeared as the top enriched GO terms.

## Discussion

### Genetics and Mapping of Root-Knot Nematode Resistance in *O. glaberrima*

*M. graminicola* is emerging as a devastating pest and affects both upland and irrigated rice causing up to 80% yield losses (Mantelin et al., 2017). It has been observed that intensely galled roots were shorter than the non-infested plants because terminal galls inhibited the roots from further elongation and prevented the plant’s root system to absorb and translocate water (Kaur, 2020). Therefore, the severity of galling has a direct consequence on plant yield. To alleviate the plant damage and yield losses, breeding for rice root-knot nematode resistance is one of the efficient and most economical strategies. With the advent of molecular markers, several nematode resistance genes and QTLs have been identified in different crop species such as tomato, potato, cotton, sugarbeet, and cowpea (Cai et al., 1997; Vander-Vossen et al., 2000; Wang et al., 2006; Wu et al., 2009; Ndeve et al., 2018).

In the past, most studies reported that rice root-knot nematode resistance was a quantitative trait (Amoussou et al., 2004; Prasad et al., 2006; Shrestha et al., 2007; Jena et al., 2013; Galeng-Lawilao et al., 2018). Here, we report that nematode resistance in *O. glaberrima* acc. IRGC102206 is under dominant monogenic control. Ideally, in BC_1_F_1_, 1:1 segregation is expected for heterozygous alleles and homozygous alleles of the recurrent parent. Due to the presence of heterozygous alleles, the trait under consideration should be moderately resistant. However, we got plants of varying resistances, from resistant to moderate resistant, suggesting that there might be non-allelic minor modifying genes that might act in an additive manner such that the phenotype is a resultant of the copy number of the alleles present in the genotype toward resistance. Similarly, the BC_2_F_2_ progenies showed wide variation for gall numbers indicating that there are a few modifying minor genes which affect the expression of a major gene to alter the resultant disease reaction. Previously, *O. glaberrima* variety CG14 has been identified as resistant to *M. graminicola* (Plowright et al., 1999; Soriano et al., 1999). But *O. glaberrima* has not been explored further to study the genetics of root-knot nematode resistance due to hybrid sterility in the interspecific crosses. However, introgression of *O. glaberrima* genes is possible by repeated backcrossing and doubled haploid breeding, even though there is a risk of losing the desirable traits from the parents (Jones et al., 1997). However, Neelam et al. (2020) have successfully transferred the bacterial blight resistance gene *xa45(t)* into Pusa 44 background and mapped the gene on chromosome 8 from *O. glaberrima* acc. IRGC102600 by backcross breeding.

So far, no information is available for rice root-knot nematode resistance mapping in African rice cultivars. To the best of our knowledge, this is the first report on the identification of a major locus and a few minor QTLs for root-knot nematode resistance using interspecific BC_1_F_1_ population derived from a cross of *O. sativa* cv. PR121 and *O. glaberrima* acc. IRGC102206 by traditional QTL mapping and BSA-seq approach. Traditional QTL mapping identified one major large effect QTL for gall number, *qGN6.1*, explaining 41% of the phenotypic variance localized in the 12.29–16.36 Mb region with increasing alleles from the donor parent. This finding was consistent with the results of BSA-seq as the major locus for nematode resistance overlapped the genomic region between 11 and 16 Mb of chromosome 6, thereby validating the existence of a major QTL for root-knot nematode resistance in this region. We also detected QTLs on chromosomes 2 and 4 with minor effects using the BSA-seq approach. Based on the BC_1_F_1_ population, we anticipated that the SNP index of the RB had a mixture of PR121 and IRGC102206 alleles, and SB had PR121 alleles for the genes/QTLs associated with nematode resistance. The SNP index of the QTL regions on chromosomes 2, 4, and 6 agreed with this expectation, but the SNP index of the QTLs on chromosomes 1, 3, 11, and 12 displayed a contrasting blueprint. Thus, the present results inferred that QTLs on chromosomes 2, 4, and 6 probably conferred the nematode resistance, whereas QTLs on chromosomes 1, 3, 11, and 12 likely contributed to the susceptibility. Identification of the higher number of genomic regions contributed by PR121 is likely because we used a backcross population having a higher proportion of recurrent parent (PR121).

Previous studies identified QTLs for root galling, eggs per total root system and eggs per gram of roots, nematode reproduction, and nematode tolerance on chromosomes 1, 3, 4, 5, 7, 8, 9, 11, and 12 (Jena et al., 2013; Dimkpa et al., 2016; Galeng-Lawilao et al., 2018). To date, there is only one report inferring that a single dominant resistance gene (*Mg1(t)*) located on chromosome 10 in Asian rice cultivar Abhishek confers resistance against *M. graminicola* (Mhatre et al., 2017). So far, the *Hsa-1Og* nematode resistance gene has been identified from *O. glaberrima* TOG5681 on the long arm of chromosome 11 between markers RM206 and RM254 that confers resistance to the cyst nematode (Lorieux et al., 2003). Lahari et al. (2019) identified genomic region from 23 Mb to the bottom of rice chromosome 11 for root-knot nematode resistance through the QTL-seq approach, which might be co-localized with the results reported by Lorieux et al. (2003). Similarly, Dimkpa et al. (2016) also identified an SNP (id11008353) associated with gall number on the long arm of chromosome 11. Also, QTLs, *qNR12.1* and *qNR12.2*, on chromosome 12 have different genomic locations for gall number–associated SNPs (Dimkpa et al., 2016). However, the BSA-seq analysis in our study identified QTL for root-knot nematode resistance on the short arm of chromosome 11 (1.01–2.38 Mb) contributed by the susceptible parent. A root-knot nematode resistance locus from 23 Mbp to the bottom of rice chromosome 11 was identified by Dimkpa et al. (2016). This might be because different sources of root-knot nematode resistance were used to identify QTL regions, as the nature of gene actions varies with the background.

Shrestha et al. (2007) identified a QTL for the gall number on the long arm of chromosome 6 flanked by R2654 and RG778 markers, explaining 9.6% of the total phenotypic variation. The results indicated that QTL, *qGN6.1/qNR6.1-6.2*, and QTL reported by Shrestha et al. (2007) were present on either sides of the centromere of chromosome 6, suggesting that gall number QTL identified in our population was controlled by a different genetic locus. QTLs/linked markers for root-knot nematode resistance identified in previous studies have been summarized in Supplementary Table S4. In the present study, one QTL for fresh root weight and two QTLs for dry shoot weight and dry root weight were identified on chromosomes 8 and 3 (two QTLs), respectively. The QTLs for dry shoot weight and dry root weight were co-localized. Earlier, the QTL for fresh root weight was mapped on chromosomes 2 and 12 (Galeng-Lawilao et al., 2018). It has to be noteworthy that in the present study, genomic regions identified for different traits through either traditional mapping or BSA-seq are unique. The major QTL could be fine mapped to identify the putative candidate gene for nematode resistance.

### Potential Roles of Candidate Genes in Root-Knot Nematode Resistance

Among the 19 candidate genes, 13 genes harbored non-synonymous mutations. Three glycosyltransferase genes were present in the *qNR*6.1 region, two of which (BGIOSGA022720 and BGIOSGA022727) had a total of 18 non-synonymous substitutions. Glycosyltransferases are involved in carbohydrate biosynthesis and cell-wall synthesis (Egelund et al., 2004). The upregulation of glycosyltransferase genes provided resistance against *Pseudomonas syringae* and *M. incognita and M. hapla* in *Arabidopsis* and tomato, respectively (Langlois-Meurinne et al., 2005; Schaff et al., 2007). However, its role in *M. graminicola* resistance is not understood currently in rice. In the present study, the peroxidase (BGIOSGA022766) gene located in *qNR6.1* plays an important role in ROS detoxification by regulating the H_2_O_2_ level and the oxidation of toxic reductants during pathogen attack. A higher level of peroxidase activity was reported in resistant genotypes of tomato during *M. incognita* infection (Chawla et al., 2013) and sweet potato (Yeon-Woo et al., 2019). Transcriptome profiling of *M. graminicola* infested rice roots showed the transcript abundance of peroxidases compared to uninfected roots (Kyndt et al., 2012). Rice root-knot nematode interactions revealed higher transcripts for nucleotide binding, catalytic, phosphatase, hydrolase, and ATPase activity after 3 and 7 days post inoculation (dpi) of *M. graminicola* (Haegeman et al., 2013).

Thioredoxin reductase has a role in ROS signaling and the protection of antioxidant enzymes to establish plant immunity during pathogen attacks. The ROS accumulation during pathogen attack results in oxidative modification of reactive free thiols in *S*-sulfenic acids (–SOH) of signaling proteins and antioxidant enzymes such as catalase (CAT). Thioredoxin reductase reduces these thiol modifications to enable ROS signaling and protection of antioxidant enzyme activities (Mata-Perez and Spoel, 2019). Similar to thioredoxin reductase, two genes of the *qNR6.2* region, acyl-coenzyme A oxidase (BGIOSGA021280) and mitogen-activated protein kinase (BGIOSGA022897), might have a potential role in signaling pathways. Acyl-CoA oxidase metabolizes the pheromone (ascaroside) secreted by plant-parasitic nematode into chemical signals through the peroxisomal β-oxidation pathway that might act as a repellent for nematodes and thus reduces infection (Manohar et al., 2020). Mitogen-activated protein kinases (MAPKs) are intracellular signaling molecules that induce a defense response against root-knot nematode. The differential expression of the MAPK genes occurred after the activation of the effector-triggered immunity (ETI) pathway in rice upon *M. graminicola* infection as reported by Hatzade et al. (2020). It has been postulated that the genes involved in defense responses, phenylpropanoid, and hormone pathways were induced in response to *M. graminicola* infection in *O. glaberrima* line TOG5681, compared to Nipponbare (Petitot et al., 2017).

In the QTL region *qNR2.1*, the kinesin-like protein gene identified has diverse roles in biotic and abiotic stresses ranging from environmental to developmental processes like cell division, cell expansion, tropisms, and hormonal signaling. Kinesin modulates the cell wall structure and function by affecting the orientation and structure of cellulose microfibrils within the cell wall (Abdelkhalek et al., 2019). Therefore, kinesin-like protein and glycosyltransferase might be involved in the synthesis and maintenance of cell wall integrity during plant–nematode interactions. Growth-regulating factors are transcription factors regulated post-transcriptionally by miRNA396 (miR396) in different plant species (Omidbakhshfard et al., 2015). The growth-regulating factors, GRF1 and GRF3, in *Arabidopsis* target a plethora of genes having roles in programmed cell death, hormone signaling, and basal defense responses during biotic stresses (Piya et al., 2020). GRF1and GRF3 control the development and differentiation of syncytia during nematode (*Heterodera schachtii*) infection in *Arabidopsis*. In the present study, we also identified GRF1 (BGIOSGA009139) in the *qNR6.2* region. The presence of non-synonymous SNPs in these candidate genes suggests their potential role for root-knot nematode resistance in rice.

## Conclusion

Understanding the genetic basis of root-knot nematode resistance and the development of functional markers to follow marker-assisted breeding is the prerequisite for nematode resistance breeding. In this study, we have identified a major QTL and two minor QTLs for *M. graminicola* resistance. Also, QTLs for fresh root weight, dry root weight, and dry shoot weight were detected on chromosomes 8 and 3, respectively. These results revealed the novel region of 11.03–16.25 Mb on chromosome 6 harboring *qNR6*.*1* and *qNR6.2* that may provide the basis for fine mapping and further exploration of novel genes associated with root-knot resistance in rice. A total of 10 and 3 candidate genes within *qNR6*.*1-qNR6.2* and *qNR2.1*, respectively, had non-synonymous SNPs that might be important for nematode resistance. The non-synonymous SNPs identified could be converted into KASP markers for effective deployment in rice breeding. The present results will increase our knowledge in understanding the molecular mechanism for root-knot nematode resistance.

## Data Availability

The datasets presented in this study can be found in online repositories. The names of the repository/repositories and accession number(s) can be found in the article/Supplementary Material.

## Supplementary Material

Supplementary Figure S1 Low quality reads **(A)** filtered through FastQC to obtain clean 856 reads **(B)** for reference alignment.
