# Supplementary material for: BSA-seq Identifies a Major Locus on Chromosome 6 for Root-Knot Nematode (Meloidogyne graminicola) Resistance From Oryza glaberrima
Source: Front Genet. 2022 Jun 14;13:871833. doi: 10.3389/fgene.2022.871833 (PMC9237506; doi:10.3389/fgene.2022.871833)
Supplement: Supplementary file 1 [file Table1.docx]

**Table S1** List of simple sequence repeats (SSR) markers analyzed and polymorphic SSR markers used in the BC_1_F_1_ population

| Chromosome number | Total number of markers analyzed | Number of polymorphic markers | Polymorphism (%) | Number of markers genotyped on BC_1_F_1_ population |
| --- | --- | --- | --- | --- |
| 1 | 53 | 27 | 50.9 | 12 |
| 2 | 53 | 24 | 45.2 | 11 |
| 3 | 52 | 25 | 48.0 | 18 |
| 4 | 45 | 17 | 37.7 | 9 |
| 5 | 39 | 11 | 28.2 | 7 |
| 6 | 40 | 10 | 25.0 | 10 |
| 7 | 43 | 7 | 16.2 | 5 |
| 8 | 43 | 9 | 20.9 | 8 |
| 9 | 31 | 8 | 25.8 | 6 |
| 10 | 37 | 13 | 35.1 | 6 |
| 11 | 37 | 6 | 16.2 | 5 |
| 12 | 39 | 3 | 7.6 | 3 |
| Total | 512 | 160 | 31.2 | 100 |

| Chromosomes | Length (bp) | Variants | Variant rate |
| --- | --- | --- | --- |
| 1 | 47,283,185 | 2,84,012 | 166 |
| 2 | 38,103,930 | 2,65,131 | 143 |
| 3 | 41,884,883 | 2,57,044 | 162 |
| 4 | 34,718,618 | 1,90,894 | 181 |
| 5 | 31,240,961 | 1,88,671 | 165 |
| 6 | 32,913,967 | 1,68,677 | 195 |
| 7 | 27,957,088 | 2,04,383 | 136 |
| 8 | 30,396,518 | 1,59,636 | 190 |
| 9 | 21,757,032 | 1,48,694 | 146 |
| 10 | 22,204,031 | 1,34,175 | 165 |
| 11 | 23,035,369 | 1,23,492 | 186 |
| 12 | 23,049,917 | 1,35,227 | 170 |
| Total | 294,434,861 | 22,60,036 | 165 |

**Table S2** Chromosome-wise distribution of variants in parents, resistant bulk and susceptible bulk identified through BSA-seq

**Table S3** Candidate genes in QTL region sassociated with rice root-knot nematode resistance identified through BSA-seq

| QTL | Chromosome | Gene locus | Start | End | Annotation |
| --- | --- | --- | --- | --- | --- |
| *qNR2.1* | 2 | BGIOSGA005507 | 34845220 | 34854243 | Kinesin like protein |
|  |  | BGIOSGA005497 | 34946498 | 34947899 | Growth regulating factor 1 |
|  |  | BGIOSGA009139 | 35056682 | 35059390 | UMP-CMP kinase |
|  |  | ENSRNA049493468 | 35262050 | 35262143 | miR164 |
|  |  | BGIOSGA005479 | 35345009 | 35346867 | Pectin esterase |
|  |  | BGIOSGA005471 | 35446440 | 35447426 | Hexosyltransferase |
|  |  | BGIOSGA005470 | 35456403 | 35457346 | Hexosyltransferase |
| *qNR3.1* | 3 | BGIOSGA012978 | 22776001 | 22779848 | Peroxidase |
|  |  | BGIOSGA010427 | 22795120 | 22796561 | Magnesium-chelatase subunit 2C |
|  |  | BGIOSGA012987 | 22930517 | 22946482 | Elongation factor 2C |
|  |  | BGIOSGA010411 | 23114071 | 23118841 | Purple acid phosphatase |
|  |  | BGIOSGA010407 | 23268808 | 23270100 | KIN17-like protein |
|  |  | ENSRNA049494981 | 23282073 | 23282137 | miR2907 |
|  |  | BGIOSGA010404 | 23313640 | 23323589 | Protein detoxification |
| *qNR3.2* | 3 | BGIOSGA010374 | 24075360 | 24077370 | Ribosomal protein L19 |
|  |  | BGIOSGA010365 | 24238553 | 24242059 | Secretory carrier associated membrane protein |
|  |  | BGIOSGA010364 | 24243032 | 24247550 | Secretory carrier associated membrane protein |
|  |  | BGIOSGA010349 | 24520427 | 24525713 | Glycosyltransferase |
|  |  | BGIOSGA010345 | 24562973 | 24570890 | Lysine t-RNA ligase |
|  |  | BGIOSGA013029 | 24579534 | 24582208 | Inositol-1-mono-phosphatase |
|  |  | BGIOSGA013038 | 24710950 | 24713763 | FRIGIDA-like protein |
|  |  | BGIOSGA013040 | 24728678 | 24729674 | FRIGIDA-like protein |
|  |  | ENSRNA049494996 | 24879143 | 24879294 | Small nucleolar RNA J26 |
|  |  | BGIOSGA013052 | 25140689 | 25141569 | Chlorophyll a-b binding protein |
| *qNR6.1* | 6 | BGIOSGA022720 | 11381748 | 11383205 | Glycosyltransferase |
|  |  | BGIOSGA021421 | 11400834 | 11402279 | Glycosyltransferase |
|  |  | BGIOSGA022727 | 11478827 | 11480266 | Glycosyltransferase |
|  |  | BGIOSGA022766 | 12402168 | 12403260 | Peroxidase |
|  |  | BGIOSGA022770 | 12472707 | 12477455 | Autophagy related protein |
|  |  | BGIOSGA022773 | 12525276 | 12528163 | Ribosomal protein L15 |
|  |  | BGIOSGA022777 | 12651761 | 12654471 | Ubiquitin E1 protein ligase |
|  |  | BGIOSGA022810 | 13505665 | 13511039 | Fructose-6-phosphate-1-phosphotransferase subunit alpha |
|  |  | BGIOSGA021328 | 13575723 | 13579551 | Thioredoxin reductase |
| *qNR6*.2 | 6 | BGIOSGA021280 | 15516716 | 15523499 | Acyl coenzyme A oxidase |
|  |  | BGIOSGA022887 | 15588056 | 15588587 | Auxin responsive protein |
|  |  | BGIOSGA022897 | 15921061 | 15929220 | Mitogen-activated protein kinase |
| *qNR11.1* | 11 | BGIOSGA034584 | 1017926 | 1018375 | 40S ribosomal protein |
|  |  | BGIOSGA034718 | 1035210 | 1035527 | Mini zinc finger protein |
|  |  | BGIOSGA034737 | 1245773 | 1248475 | S-acyl transferase |
|  |  | BGIOSGA034730 | 1272304 | 1276080 | Non-specific serine -threonine protein kinase |
|  |  | BGIOSGA034546 | 1373325 | 1376009 | Auxin efflux carrier component |
|  |  | BGIOSGA034744 | 1383541 | 1385189 | WAT1 related protein |
|  |  | BGIOSGA034541 | 1417336 | 1420014 | Auxin efflux carrier component |
|  |  | BGIOSGA034540 | 1421026 | 1425580 | U3 smaller nucleolar ribonucleoprotein |
|  |  | BGIOSGA034525 | 1661244 | 1662444 | Sulfotransferase |
|  |  | BGIOSGA034524 | 1664203 | 1665219 | Sulfotransferase |
|  |  | BGIOSGA034794 | 1965216 | 1968838 | Peptidyl propyl isomerase |
|  |  | BGIOSGA034500 | 1975031 | 1978699 | Pyruvate kinase |
| *qNR12.1* | 12 | ENSRNA049493019 | 10691630 | 10691702 | tRNA-Ala for anticodon AGC |
|  |  | ENSRNA049492915 | 10735722 | 10735794 | tRNA-Ala for anticodon AGC |
|  |  | BGIOSGA037316 | 10833893 | 10842526 | Endoglucanase |
|  |  | BGIOSGA036295 | 10920958 | 10938547 | Beta-galactosidase |
|  |  | BGIOSGA036274 | 11485957 | 11486567 | Probable histone H2A.8 |
|  |  | BGIOSGA036270 | 11551444 | 11552054 | Probable histone H2A.7 |
|  |  | BGIOSGA036265 | 11635369 | 11637929 | Chloride channel protein |
|  |  | BGIOSGA036259 | 11969221 | 11975013 | Bifunctional dihydrofolate reductase thymidylate synthase |
|  |  | BGIOSGA037342 | 12433622 | 12435064 | UDP-glucose-6-dehydrogenase |
|  |  | BGIOSGA037341 | 12426192 | 12427634 | UDP-glucose-6-dehydrogenase |
| *qNR12.2* | 12 | BGIOSGA036039 | 17817776 | 17819260 | Patatin |
|  |  | BGIOSGA037557 | 17910874 | 17912400 | Patatin |
|  |  | BGIOSGA037558 | 17915254 | 17917074 | Xylose isomerase |
|  |  | BGIOSGA037562 | 17960570 | 17962407 | Patatin |
|  |  | BGIOSGA037563 | 17990726 | 17992340 | Protein detoxification |
|  |  | ENSRNA049494737 | 18044897 | 18044982 | Small nucleolar RNA Z159/U59 |
|  |  | ENSRNA049494733 | 18045410 | 18045508 | Small nucleolar RNA Z159/U59 |
|  |  | BGIOSGA037569 | 18106591 | 18107369 | Pathogenesis related protein |
|  |  | BGIOSGA036022 | 18186487 | 18192232 | CTP Synthase |
|  |  | BGIOSGA036014 | 18367286 | 18372540 | Lipoxygenase |
|  |  | BGIOSGA037588 | 18415067 | 18421384 | Lipoxygenase |
|  |  | BGIOSGA037589 | 18424656 | 18434305 | Lipoxygenase |
|  |  | BGIOSGA037590 | 18444086 | 18444994 | DNA-directed RNA polymerase subunit |
|  |  | BGIOSGA036008 | 18451670 | 18456504 | DNA replication licensing factor MCM7 |
|  |  | BGIOSGA036005 | 18496168 | 18497534 | Glycosyltransferase |
|  |  | BGIOSGA037593 | 18518398 | 18525782 | Genomes uncoupled1 |
|  |  | BGIOSGA037594 | 18529938 | 18534078 | Phosphoinositide phospholipase C |
|  |  | BGIOSGA036000 | 18610296 | 1861335 | Pectinesterase |
|  |  | BGIOSGA037614 | 18832998 | 18836921 | Putative diamino pimelate epimerase %2C chloroplastic |
|  |  | BGIOSGA037616 | 18885532 | 18886548 | CASP-like protein |
|  |  | BGIOSGA035979 | 18905862 | 18906502 | Metallothionein-like protein 1 |
|  |  | BGIOSGA037618 | 18914717 | 18915372 | CASP like protein |
|  |  | ENSRNA049494744 | 18965667 | 18965774 | Small nucleolar RNA Z194 |
|  |  | ENSRNA049494742 | 18967500 | 18967603 | Small nucleolar RNA Z194 |
|  |  | BGIOSGA035977 | 18989951 | 18994416 | RING-type E3 ubiquitin transferase |
|  |  | ENSRNA049493001 | 19025023 | 19025095 | tRNA-lys for anticodon CUU |
|  |  | BGIOSGA037630 | 19026871 | 19027444 | Metallothionein like protein 1 |
|  |  | BGIOSGA035969 | 19162215 | 19163029 | rRNA-glycosidase |
|  |  | BGIOSGA035968 | 19178865 | 19179680 | rRNA-glycosidase |
|  |  | ENSRNA049494740 | 19245303 | 19245606 | Eukaryotic large subunit ribosomal RNA |
|  |  | BGIOSGA035957 | 19306125 | 19399340 | Purple acid phosphatase |
|  |  | BGIOSGA035956 | 19311427 | 19315375 | Purple acid phosphatase |
|  |  | BGIOSGA035955 | 19320015 | 19323143 | Purple acid phosphatase |
|  |  | ENSRNA049492950 | 19324483 | 19324555 | tRNA-lys for anticodon CUU |
|  |  | BGIOSGA035954 | 19324972 | 19328527 | Diacylglycerol kinase |
|  |  | ENSRNA049494739 | 19373070 | 19373172 | Eukaryotic small subunit rRNA |
|  |  | ENSRNA049494735 | 19373221 | 19373357 | Eukaryotic small subunit rRNA |
|  |  | BGIOSGA035949 | 19459328 | 19462053 | Hexosyltransferase |
|  |  | BGIOSGA035948 | 19474636 | 19478619 | Hexosyltransferase |
|  |  | BGIOSGA037691 | 20004843 | 20009925 | Calcium transporting ATPase |
|  |  | BGIOSGA035918 | 20123046 | 20125377 | Cytoplasmic tRNA 2-thiolatin protein |
|  |  | ENSRNA049492953 | 20573222 | 20573295 | tRNA-Glu for anticodon UUC |
|  |  | BGIOSGA037741 | 20859473 | 20860460 | Auxin responsive protein |
|  |  | BGIOSGA037750 | 21063685 | 21073223 | Protein root hair defective 3 homolog |
|  |  | BGIOSGA035880 | 21073422 | 21076878 | Serine/threonine protein kinase Nek2 |
|  |  | BGIOSGA035857 | 21401243 | 21402430 | CASP-like protein |
|  |  | BGIOSGA035845 | 21551315 | 21556978 | Homeobox-leucine zipper protein HOX33 |
|  |  | BGIOSGA035840 | 21626023 | 21630818 | Auxin response factor |
|  |  | BGIOSGA037790 | 21679775 | 21681199 | Protein kinase |
|  |  | BGIOSGA037797 | 21741994 | 21744165 | Protein detoxification |
|  |  | BGIOSGA035830 | 21758033 | 21762665 | Kinesin like protein |
|  |  | BGIOSGA037799 | 21783751 | 21784644 | Transcription factor PCF8 |
|  |  | BGIOSGA035823 | 21927220 | 21935111 | Serine threonine protein phosphatase |
|  |  | BGIOSGA035799 | 22331167 | 22335544 | Cysteine synthase |
|  |  | BGIOSGA035796 | 22374783 | 22377077 | Deoxypurine hydroxylase |
|  |  | BGIOSGA037845 | 22476024 | 22478990 | Methionine aminopeptidase 2 |
|  |  | ENSRNA049492974 | 22563123 | 22563196 | tRNA-Glu for anticodon UUC |
|  |  | ENSRNA049492969 | 22624761 | 22624832 | tRNA-Glu for anticodon UUC |
|  |  | BGIOSGA035779 | 22648517 | 22650757 | rRNA-glycosidase |
|  |  | BGIOSGA035778 | 22662292 | 22666583 | Malate dehydrogenase |
|  |  | ENSRNA049492965 | 22780501 | 22780573 | tRNA-Ala for anticodon UGC |
|  |  | BGIOSGA035761 | 22855405 | 22856793 | Purple acid phosphatase |
|  |  | BGIOSGA035760 | 22858321 | 22861104 | Purple acid phosphatase |
|  |  | BGIOSGA035759 | 22864177 | 22867021 | Purple acid phosphatase |
|  |  | BGIOSGA037870 | 22942235 | 22948308 | Plasma membrane ATPase |

| Chromosome | Linked Marker/Marker interval (Mb) | References |
| --- | --- | --- |
| 2 | C601-RG256 (30.27-33.93) | Shreshta et al.,2007 |
| 3 | id3004493-id3011085 (8.54-8.59) | Dimkpa et al., 2016 |
| 4 | id4000274-id4001733 (0.47-4.27)  K_id4001113-id4004802 (~7.89)  K_id4010924-id4011683 (>26.85) | Dimkpa et al., 2016  Lawilao et al., 2018 |
| 6 | R2654-RG778 (22.87-26.23) | Shreshta et., 2007 |
| 11 | RZ141-G320 (4.07-6.68)  id11008353 (22.2)  QTL-seq (23.0-29.0 Mb) | Shreshta et., 2007  Dimkpa et al., 2016  Lahari et al., 2019 |
| 12 | id12005724-id12005794 (16.90-17.28) | Dimkpa et al., 2016 |

**Table S4** QTLs conferring root-knot nematode resistance identified in previous studies on different chromosomes
