# Supplementary figures and images for: BSA-seq Identifies a Major Locus on Chromosome 6 for Root-Knot Nematode (Meloidogyne graminicola) Resistance From Oryza glaberrima
Source: Front Genet. 2022 Jun 14;13:871833. doi: 10.3389/fgene.2022.871833 (PMC9237506; doi:10.3389/fgene.2022.871833)

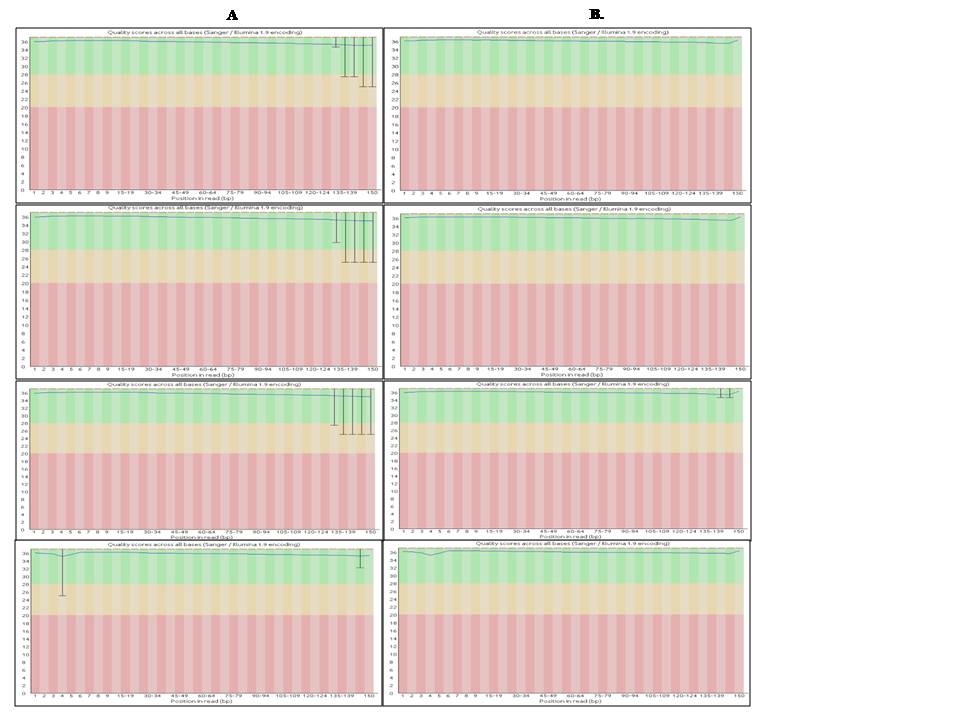

Supplement: Supplementary file 2 [file Image1.JPEG]
